# Motion resistance in peripheral oxygen saturation monitoring using Biolight Analog SpO_2_ compared to Masimo SpO_2_: a non-inferiority study

**DOI:** 10.1186/s12871-024-02823-z

**Published:** 2024-11-27

**Authors:** Ting Yang, Yong Liu, FengHua Cai, Yong Li, Muhammad Saqib Mudabbar

**Affiliations:** 1 Department of Anesthesiology, Affiliated Hospital of Southwest Jiaotong University, The Third People’s Hospital of Chengdu, Chengdu, Sichuan 610014 China; 2 Department of Clinical Applications, Guangdong Biolight Meditech Co., Ltd., No.2 Innovation 1st Road, Technical Innovation Coast, Hi-tech Zone, Zhuhai, Guangdong 519085 China; 3 Department of Linguistics, Central China Normal University, 152 Luyu Road, Hongshan District, Wuhan City, Hubei Province 430079 China; 4 Department of Biotechnology, Shanghai Ocean University, No.999, Huchenghuan Rd, Nanhui New City, Shanghai 201306 China; 5 Department of Electronics Engineering, Heilongjiang University, No.74, Xuefu Road, Nangang District, Harbin City, Heilongjiang province 150080 China; 6 Department of Research and Development, Guangdong Biolight Meditech Co., Ltd., No.2 Innovation 1st Road, Technical Innovation Coast, Hi-tech Zone, Zhuhai, Guangdong 519085 China; 7 Department of Cardiovascular Medicine, The Affiliated Hospital of Southwest Medical University, Luzhou, Sichuan 646000 China

**Keywords:** Pulse oximetry, Motion resistance, Analog SpO_2_, Healthy adults, Monitoring, Accuracy, Alarms, “Oximetry/instrumentation”[Mesh]

## Abstract

**Background:**

Pulse oximeters are vital for assessing blood oxygen levels but can produce inaccurate readings during patient motion, leading to false alarms and alarm fatigue. Analog SpO_2_ Technology, which uses analog waveforms to filter motion artifacts, may improve accuracy compared to digital sensors. However, the effectiveness of this technology in reducing false alarms in clinical settings remains unclear. This study assesses and compares the motion resistance of Analog SpO_2_ Technology of two devices in the market.

**Methods:**

Thirty healthy adults underwent controlled experiments (Control, Linear Motion, Angular Motion) using two pulse oximeters. Linear Motion tested hand displacement impact, while Angular Motion involved rhythmic hand motions at 120 bpm and 160 bpm.

**Results:**

Both devices performed similarly in Control, with no disruptions. In Linear Motion, mild disruptions occurred, but no significant differences in SpO_2_ readings or alarms. Angular Motion at 120 bpm showed stability with no alarms. At 160 bpm, Device B (Biolight Analog SpO_2_) had fewer technical alarms but more SpO_2_ alarms than Device A (Masimo Analog SpO_2_).

**Conclusions:**

Analog SpO_2_ exhibited motion resistance under static, linear and continuous waving angular motion up to 120 bpm and 160 bpm, but alarms occurred at 160 bpm with continuous tapping angular motion. These findings signify non-inferiority of either device in clinical settings. Further studies should include patients with cardiovascular and/or respiratory diseases.

**Trial Registration:**

The study was submitted to and approved by the Biolight Ethics Committee (S0723), and written informed consent from all participants was obtained.

## Graphical abstract

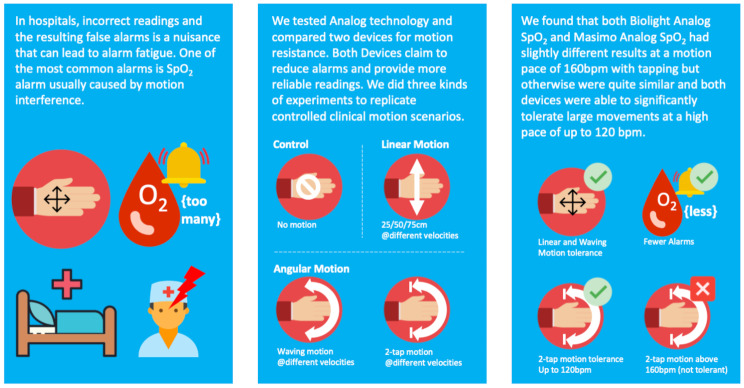

## Introduction

A pulse oximeter is a non-invasive tool used to measure arterial blood oxygen saturation [1]. It works by shining light through the skin with its photodiodes and detecting it with a sensor that records light absorption as a function of time and can measure the percentage of hemoglobin bound to oxygen [2]. This lets healthcare providers assess the patient’s oxygenation status, making pulse oximetry one of the most important devices in the medical industry [3]. For patients of all ages, healthcare providers rely on these devices to diagnose hypoxemic events and observe treatment responses, which helps them make informed clinical decisions [4–11]. The pulse oximeters are useful devices, but sometimes their values are not very accurate, which can be due to a number of factors, including motion [12]. Previous studies have shown that motion has caused SpO_2_ (SpO_2_ = peripheral oxygen saturation) readings to be lower than actual readings and caused unnecessary alarms [13–15]. These alarms can lead to alarm fatigue, hindering attention when required. Engineers and researchers have worked together to remove these motion artifacts, which claim them to be very effective [16, 17]. Analog SpO_2_ technology measures and transmits the data in analog waveforms, which allows the devices to filter motion artifacts with proprietary algorithms and have proven to be better than digital sensors [17]. Even though this technology has existed for a while, newer publications on alarm fatigue still mention lots of false alarms in clinical settings, including technical and non-actionable alarms [18]. These alarms can be caused by many monitored parameters. Whether these alarms are caused by Analog SpO_2_ Technology or lack of adaptation of this technology is unknown. We hypothesize that Analog SpO_2_ technology could be more resistant to motion and thus decrease false alarms. The objective of this study was to assess and compare the extent of motion resistance of Analog SpO_2_ Technology among devices that currently offer this technology.

## Method

### Patient characteristics

This was a single-center, experimental study performed at the laboratory of Biolight from March 30, 2023 to April 21, 2023. In this study, we recruited a total of 30 adult subjects (15 males and 15 females) with ages ranging from 24 to 52 years. All participants were adults and in good health with various skin-tones, with no known cardiovascular or respiratory conditions, as that could significantly affect their SpO_2_ readings, and would have made it difficult to distinguish the cause of alarms, as this study’s objective was to assess motion resistance. Additionally, participants were required to abstain from consuming caffeine or alcohol for at least 12 h prior to the experiments to minimize potential confounding factors. Measurements were taken under these controlled environmental conditions to minimize the impact of extraneous factors on SpO_2_ readings.

### Ethics approval and consent to participate

The study was submitted to and approved by the Biolight Ethics Committee (S0723), and written informed consent from all participants was obtained.

### Sample size calculation

The minimal sample size was calculated according to our unpublished preliminary study, which showed that movement caused a 34% chance of SpO_2_ alarms with digital SpO_2_ devices used in the hospital compared to 8% alarms with Device A and Device B. We set α = 0.05, 1-β = 0.9, and used the “Sample Size Calculator” to calculate the sample size online [19]; the total sample size was 24. Considering the loss to follow-up rate of 25%, it was estimated that 30 cases need to be included.

### Equipment used

1. Biolight AnyView P22 Patient Monitor (Patient Monitor, Guangdong Biolight Meditech Co., Ltd., Zhuhai, People’s Republic of China).
2. Biolight SpO_2_ module (Module for Patient Monitor, Guangdong Biolight Meditech Co., Ltd., Zhuhai, People’s Republic of China).
3. Masimo SpO_2_ module (Module for Patient Monitor, Masimo Corporation, Irvine, USA).
4. Biolight adult reusable analog SpO_2_ sensor 15-199-0359 (Accessory for Patient Monitor, Guangdong Biolight Meditech Co., Ltd., Zhuhai, People’s Republic of China).
5. Masimo SpO_2_ adult reusable Finger Clip Sensor RD SET DCI REF 4050 (Accessory for Patient Monitor, Masimo Corporation, Irvine, USA).
6. Masimo RD Rainbow SET MD20-12 Patient cable REF 4073(Accessory for Patient Monitor, Masimo Corporation, Irvine, USA).
7. 120 cm Camera railing with slider and camera or phone mounting attachments (Camera Railing and mount, Sutesheyingqicai Company, Shenzhen, China).
8. Apple iPhone XS Max (with Metronome App and Camera App) (Apple, Cupertino, USA).
9. Zip ties (Shaodeng Plastic Company, Leqing, China).
10. Paper Scissors (Deli, Ninghai, China).
11. Surgical tape (Haishi, Qingdao, China).
12. Soft Velcro cable management tape (Xinhui, Shanghai, China).
13. 1-meter-long centimeter-scale ruler (Xiaobangshou, Suzhou, China).
14. Large geometry set protractor (Jinsihuo, Taizhou, China).

### Outcomes

**Fig. 1:**
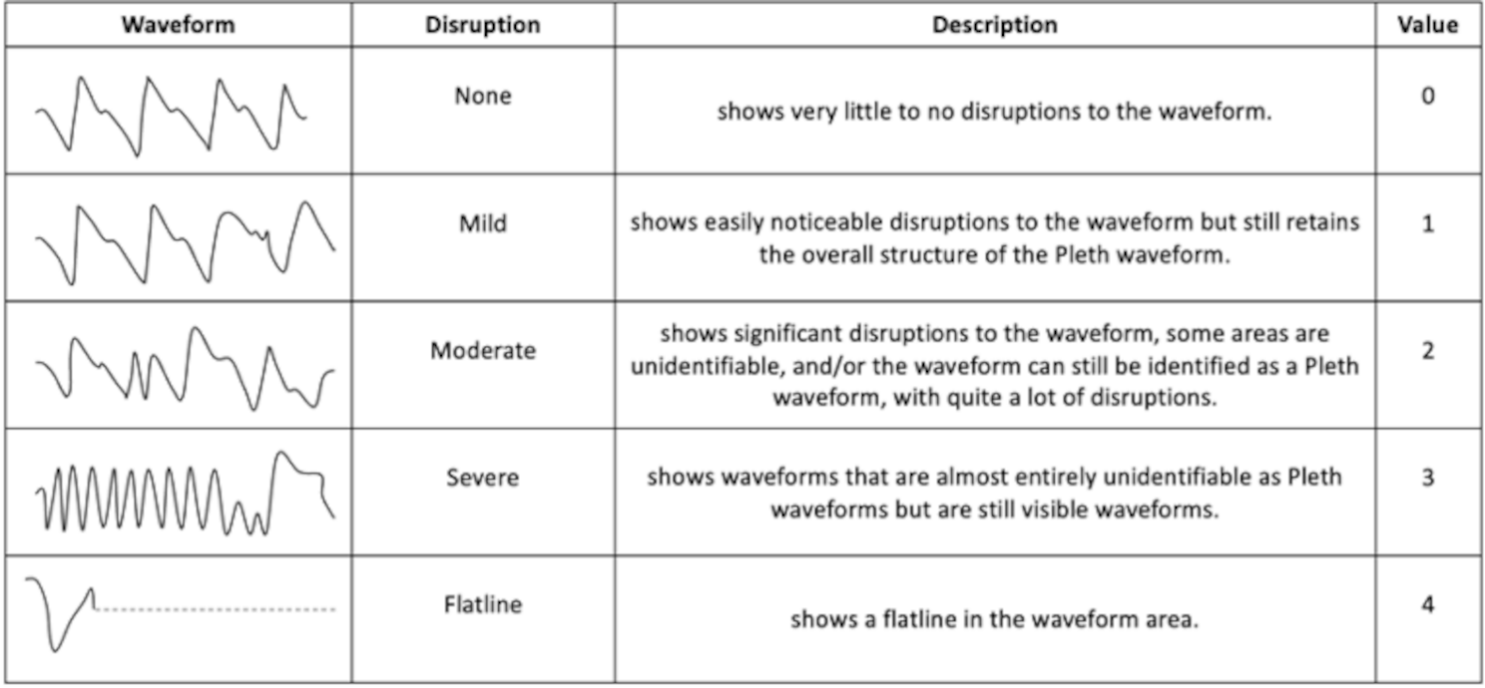
Shows the description of disruptions in plethysmographic waveforms

Technical and SpO_2_ alarms were considered the primary outcome, and SpO_2_ Values and Disruptions *see* Fig. 1 in Plethysmography Waveforms were considered secondary outcomes. Disruptions in Plethysmography Waveforms were defined as none, mild, moderate, severe, and flatline. The acceptable thresholds of non-inferiority were determined by not sounding significantly more alarms, and not having a significantly large difference in SpO_2_ values or waveform disruptions. This significance is equivalent to statistical significance.

### Statistical analysis

The differences in the primary and the secondary outcomes were assessed for significance by comparing means between the results of the two devices, one-sided *p* values were considered. The differences were considered significant if *p* < 0.05. The effect size (Cohen’s d) for the comparison of the two devices was calculated to quantify the magnitude of the difference, with a value of 0.2 indicating a small effect. To assess agreement between the two devices; Bland-Altman plot was generated using the SpO_2_ percentage values, from the Angular Motion experiment at 120 bpm and 160 bpm with both waving and 2-tap motion data separately, of the two devices. Statistical analyses were performed with SPSS 26.0 (IBM, Chicago, IL, USA).

### Procedure

We performed three experiments: a Control Experiment, a Linear Motion Experiment, and an Angular Motion Experiment.

### Control experiment

In the control group, SpO_2_ and the appearance of Plethysmography waveforms were monitored for 10 min on both Device A (Masimo Analog SpO_2_) and Device B (Biolight Analog SpO_2_) while keeping the hand still. First, Device A’s Probe was placed on the middle finger, and Device B’s Probe was placed on the index finger. After 5 min, the probes were switched; Device B’s Probe was placed on the middle finger, and Device A’s Probe was placed on the index finger.

### Linear motion experiment

SpO_2_ readings during the Linear Motion Experiment included static readings and dynamic readings. Static readings were taken right before the hand was displaced when readings stabilized. The dynamic readings were the lowest readings within 15 s after the motion.

Two types of alarms were recorded: SpO_2_ alarms, the lower limit set was 90%, and technical alarms. If the alarm sounded within 15 s after the motion, it was recorded as a yes; otherwise, it was recorded as a no. This was later translated into statistical values: a yes translated into a 1, and a no translated into a 0.

In the Liner Motion Experiment, there were three groups. The groups were named “25 cm”, “50 cm”, and “75 cm”. In each of these groups, participants moved their hands with Biolight’s and Masimo’s probes attached for 25 cm, 50 cm, and 75 cm, respectively. To ensure that they all moved their hands at the same velocity, we used a camera railing with a heavy slider to calibrate the velocity and be used as a visual reference. The slider moved under its weight when the camera railing was placed at an inclined angle. This slider’s motion was taken as reference and the participants moved their hands along with the slider without touching it to match its speed.

The displacement was adjusted by measuring two points on the railing with a ruler and marking them with zip ties. The velocity was increased by increasing the height of one end of the railing. This height was determined by the angle of the inclined railing. This angle was set using a protractor. A video of the camera mount sliding down the railing was recorded using the phone’s camera. This was done to calculate the velocity by counting frames that showed motion of the camera mount between the two marked points. The velocity was confirmed by repeating the motion and recording it three times, and the most reliable readings of velocity were recorded.

**Fig. 2:**
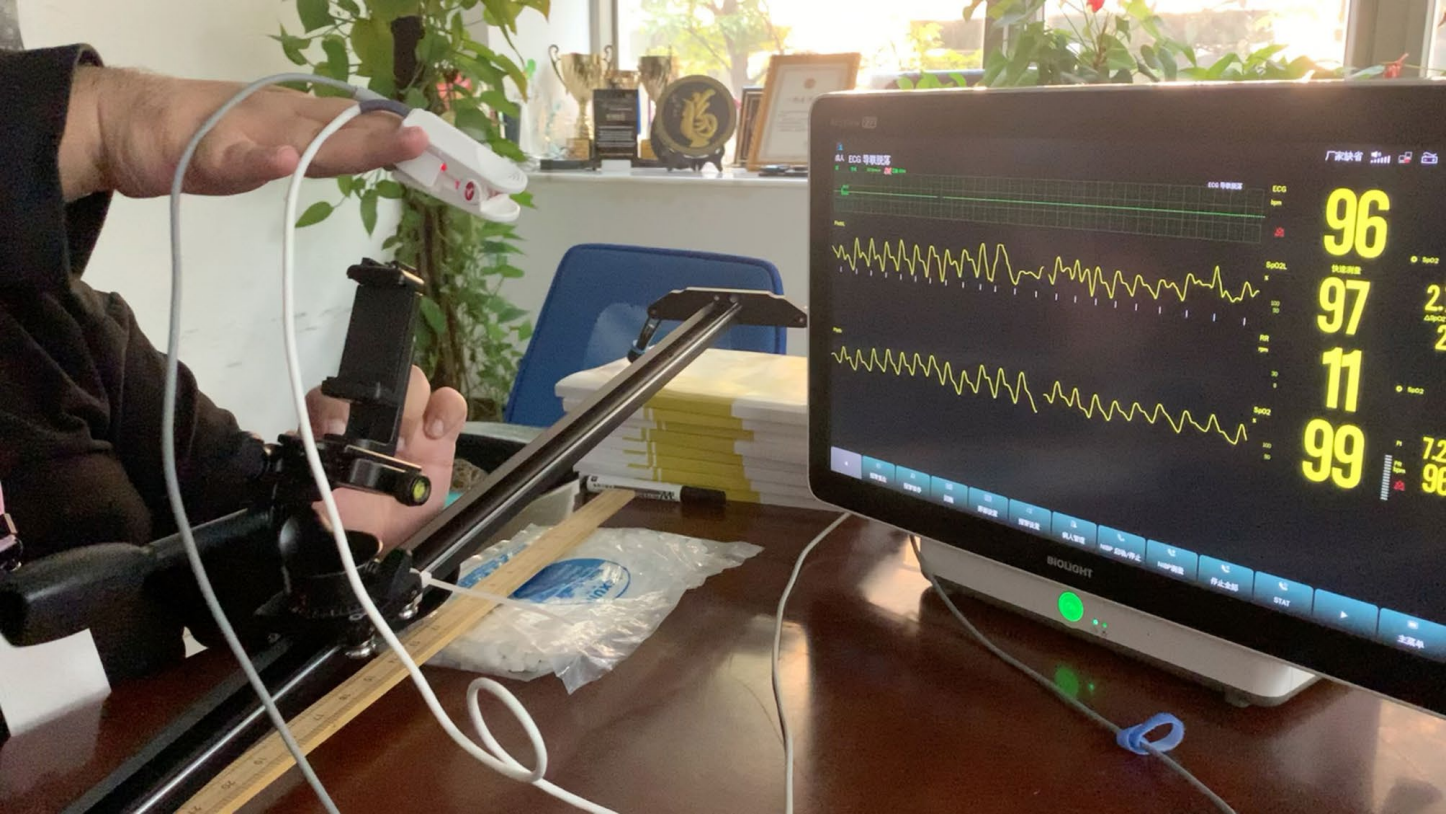
Linear Motion Experiment Setup. The left side shows a camera railing with a slider attached and the researcher’s hand hovering above it with a Biolight SpO_2 _sensor on the index finger and a Masimo SpO_2_ sensor on the middle finger. The patient monitor on the right is a Biolight Anyview P22 patient monitor, displaying Masimo SpO_2_ above (label on screen: SpO2L) and Biolight SpO_2_ below (label on screen: SpO2)

After the motion, to avoid any unrelated alarms, the hand was kept steady for another 15 s to observe for any changes in the waveform or alarms sounded, and the lowest SpO_2_ readings within 15 s of the motion were recorded. Each length and velocity subset were repeated 10 times and readings were recorded. Device A and Device B probes were placed on the middle and index fingers, respectively, for the first five readings, and for the next five readings, the placement of the probes was switched. See Fig. 2.

### Angular motion experiments

**Fig. 3:**
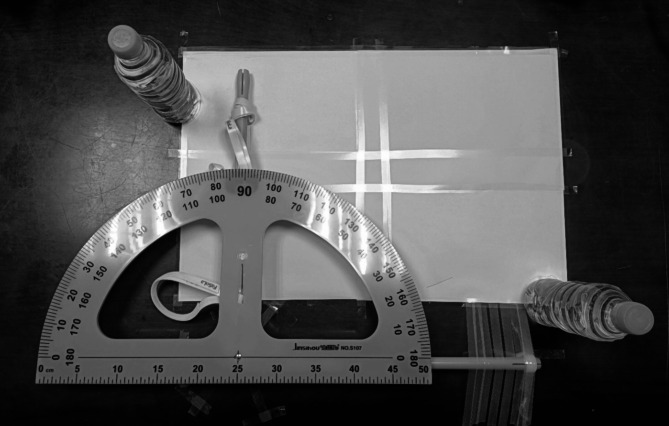
Angular Motion Experiment Setup. A large compass is tapped to a flat table surface with a protractor placed right on its pivot point, marking a 90° angle. There are two Velcro ribbons firmly attached to the compass’s left shaft to secure the arm to it. A paper sheet was placed on the surface to reduce friction, and water bottles were placed to mark the boundaries

In this experiment, the participants moved their arms in an angular motion. To keep the motion consistent among all the participants, their arms were tied to a large compass with Velcro ribbons attached to it to secure the arm in place and allow for a consistent angular motion of 90*°* at the pivot point. See Fig. 3.

The participants followed a metronome app that sounded a continuous rhythm of 120 bpm and 160 bpm. Each pace was considered a subgroup, 120 bpm and 160 bpm subgroups. We choose rhythms of 120 bpm and 160 bpm for this experiment because in our preliminary experiment we had 4 subgroups of rhythms 40 bpm, 80 bpm, 120 bpm and 160 bpm; there was no statistical difference in the 40 bpm, 80 bpm and 120 bpm subgroups. Therefore, we chose the fastest rhythm among these to be tested against 160 bpm. The data was recorded by the monitor, and timestamps were recorded on paper to later access the data from the monitor. The trend data for the Angular Motion Experiments was exported directly from the monitor with the help of the software engineer and was then filtered and classified by timestamps. The filtration involved removing empty data cells when the monitor was not making readings during rest or between participants. Filtration also meant only keeping the 60 s of data during the experiment; any values after the experiment were removed. The data was in intervals of 5 s. So, there were 12 readings for each subgroup.

### Waving motion group

In the Waving Motion group, the participants waved their hands, rotating at the elbows by about 90 degrees in a rhythmic motion for 60 s at 120 bpm and then for another 60 s at 160 bpm. However, the actual movement of the hand was slightly less than 90 degrees since 90 degrees was a bit uncomfortable for participants to do. Two areas were marked on the table where the participants could float their hands over while keeping their elbows on the table. The participants were asked to rest for 3 min before performing each subgroup experiment.

### 2-tap motion group

In the 2-Tap Motion group, the process was the same as the Waving Motion group, except that with each beat, the participants had to softly tap on the surface of the table along with the waving motion.

### Confounding factors

To control for potential external influences, all measurements were conducted under standardized conditions. Subjects were instructed to abstain from caffeine, alcohol, and medications that could affect cardiovascular or respiratory function before the experiment. Each subject was given time to rest upon arrival to minimize physiological variation due to nervousness or recent activity. To prevent light interference, room lights were turned off, and temperature was main tained at a consistent level for all trials. The fingers on which the probes were connected were also switched in a way that each probe was on each of the two fingers for 50% of the time. Participants were intentionally selected to include varied skin tones, reflecting the device’s applicability across diverse real-world populations.

## Results

We recruited a total of 30 participants; however, 2 participants were unable to comprehend the instructions clearly and randomly stopped in the middle of the experiment or started talking. Therefore, their results were omitted, and 4 participants who participated later changed their minds and revoked their consent to let us publish their experimental results.

The results were consistent across all participants, suggesting that the control measures effectively minimized variability. Furthermore, the inclusion of participants with varied skin tones supports the device’s robustness and reliability across a diverse population.

According to the results of The Control Experiment, there were no waveform disruptions, no technical or SpO_2_ alarms, and the SpO_2_ values of both Device A and Device B had no significant differences regardless of the finger the sensor was attached to. See Table 1.

**Table 1:** Control experiment. Data is shown as mean ± standard deviation (*n* = 24)

Static Monitoring								
Finger	Device A^1^ Waveform Disruption (0–4)	Device B^2^ Waveform Disruption (0–4)	Device A SpO_2_ (%±S)	Device B SpO_2_ (%±S)	Device A Technical Alarms (*n* ± S)	Device B Technical Alarms (*n* ± S)	Device A SpO_2_ Alarms (*n* ± S)	Device B SpO_2_ Alarms (*n* ± S)
Index (x̄±s)	0 ± 0	0 ± 0	99.3 ± 0.6	99.8 ± 0.4	0 ± 0	0 ± 0	0 ± 0	0 ± 0
Middle (x̄±s)	0 ± 0	0 ± 0	99.2 ± 0.7	99.6 ± 0.5	0 ± 0	0 ± 0	0 ± 0	0 ± 0

^1^Device A = Masimo Analog SpO_2_, ^2^Device B = Biolight Analog SpO_2_

According to the linear motion experiment data, as the displacement increased from 0.25 m to 0.75 m, the waveform disruption increased from 0.26 to 1.02 for Device A and 0.36 to 0.68 for Device B. The mean waveform disruption of Device A and Device B was 0.65 and 0.53, respectively. Both indicate None to Mild waveform disruptions. Static and Dynamic SpO_2_ values of both Device A and Device B were similar, with no significant differences. No technical or SpO_2_ alarms were sounded by either Device A or Device B throughout this experiment. See Table 2.

**Table 2:** Linear Motion Experiment. Data is shown as mean ± standard deviation (*n* = 24)

**Linear Motion in terms of Displacement Value**								
**Displacement (m)**	**Device A**^**1**^ **Waveform Disruption (0–4)**	**Device B**^**2**^ **Waveform Disruption (0–4)**	**Device A SpO**_**2**_ **(%±S)**	**Device B SpO**_**2**_ **(%±S)**	**Device A Technical Alarms (*n*** ± **S)**	**Device B Technical Alarms (*n*** ± **S)**	**Device A SpO**_**2**_ **Alarms (*n*** ± **S)**	**Device B SpO**_**2**_ **Alarms (*n*** ± **S)**
0.25 m (x̄±s)	0.3 ± 0.4	0.4 ± 0.6	99.3 ± 0.7	99.6 ± 0.5	0 ± 0	0 ± 0	0 ± 0	0 ± 0
0.5 m (x̄±s)	0.7 ± 0.7	0.5 ± 0.6	99.4 ± 0.6	99.9 ± 0.3	0 ± 0	0 ± 0	0 ± 0	0 ± 0
0.75 m (x̄±s)	1 ± 0.9	0.7 ± 0.7	99.5 ± 0.5	99.8 ± 0.4	0 ± 0	0 ± 0	0 ± 0	0 ± 0
**Linear Motion in terms of Finger used**								
**Finger**	**Device A Waveform Disruption (0–4)**	**Device B Waveform Disruption (0–4)**	**Device A SpO**_**2**_ **(%±S)**	**Device B SpO**_**2**_ **(%±S)**	**Device A Technical Alarms (*n*** ± **S)**	**Device B Technical Alarms (*n*** ± **S)**	**Device A SpO**_**2**_ **Alarms (n ± S)**	**Device B SpO**_**2**_ **Alarms (n ± S)**
Index (x̄±s)	0.5 ± 0.7	0.5 ± 0.7	99.4 ± 0.6	99.8 ± 0.4	0 ± 0	0 ± 0	0 ± 0	0 ± 0
Middle (x̄±s)	0.8 ± 0.8	0.5 ± 0.6	99.4 ± 0.6	99.7 ± 0.5	0 ± 0	0 ± 0	0 ± 0	0 ± 0
**Linear Motion in terms of Velocity**								
**Velocity (m/s)**	**Device A Waveform Disruption (0–4)**	**Device B Waveform Disruption (0–4)**	**Device A SpO**_**2**_ **(%±S)**	**Device B SpO**_**2**_ **(%±S)**	**Device A Technical Alarms (*n*** ± **S)**	**Device B Technical Alarms (*n*** ± **S)**	**Device A SpO**_**2**_ **Alarms (n ± S)**	**Device B SpO**_**2**_ **Alarms (n ± S)**
0.26	0.2 ± 0.4	0.1 ± 0.3	98.9 ± 0.3	99.6 ± 0.7	0 ± 0	0 ± 0	0 ± 0	0 ± 0
0.31	0.6 ± 0.5	0.9 ± 0.7	100 ± 0	100 ± 0	0 ± 0	0 ± 0	0 ± 0	0 ± 0
0.32	0.7 ± 0.5	0.9 ± 0.7	99.3 ± 0.5	99.9 ± 0.3	0 ± 0	0 ± 0	0 ± 0	0 ± 0
0.33	1 ± 0.8	0.7 ± 0.7	99.6 ± 0.5	99.8 ± 0.4	0 ± 0	0 ± 0	0 ± 0	0 ± 0
0.37	0.3 ± 0.5	0.1 ± 0.3	98.9 ± 0.6	99.3 ± 0.5	0 ± 0	0 ± 0	0 ± 0	0 ± 0
0.4	1.3 ± 0.8	0.6 ± 0.7	99.4 ± 0.5	99.8 ± 0.4	0 ± 0	0 ± 0	0 ± 0	0 ± 0
0.43	1.5 ± 1	0.2 ± 0.4	99 ± 0	99.8 ± 0.4	0 ± 0	0 ± 0	0 ± 0	0 ± 0
0.45	0.2 ± 0.4	0.6 ± 0.7	98.5 ± 0.5	99.3 ± 0.5	0 ± 0	0 ± 0	0 ± 0	0 ± 0
0.47	0 ± 0	0.1 ± 0.3	100 ± 0	99.9 ± 0.3	0 ± 0	0 ± 0	0 ± 0	0 ± 0
0.5	0.6 ± 0.7	0.5 ± 0.5	99.1 ± 0.6	100 ± 0	0 ± 0	0 ± 0	0 ± 0	0 ± 0
0.52	0.8 ± 0.4	0.8 ± 0.8	99.6 ± 0.5	100 ± 0	0 ± 0	0 ± 0	0 ± 0	0 ± 0
0.58	0.2 ± 0.4	0.3 ± 0.5	99.4 ± 0.7	99.8 ± 0.4	0 ± 0	0 ± 0	0 ± 0	0 ± 0
0.65	0.6 ± 0.8	0.4 ± 0.7	99.9 ± 0.3	99.9 ± 0.3	0 ± 0	0 ± 0	0 ± 0	0 ± 0
0.66	1 ± 1.3	0.8 ± 0.9	99.6 ± 0.5	99.6 ± 0.5	0 ± 0	0 ± 0	0 ± 0	0 ± 0
0.71	0.8 ± 0.6	0.9 ± 0.6	99.9 ± 0.3	99.9 ± 0.3	0 ± 0	0 ± 0	0 ± 0	0 ± 0

^1^Device A = Masimo Analog SpO_2_, ^2^Device B = Biolight Analog SpO_2_

With respect to velocity, the waveform disruptions peaked at 1.5 for Device A, which is between the Mild to Moderate range, and at 0.9 for Device B, which is between the None to Mild range. The increase in disruptions did not show a clear association with velocity. For Device A, the waveform disruptions increased with a velocity of up to 0.43 m/s; however, it went back down to 0.2 at 0.45 m/s and all the way to 0.00 at 0.47 m/s and then went back up to 1.00 at 0.66 m/s. For Device B, the waveform disruptions were higher at both lower and higher velocities of 0.31 m/s, 0.32 m/s, 0.33 m/s, 0.52 m/s, 0.66 m/s, and 0.77 m/s while lower at other velocities. See Table 2.

**Fig. 4:**
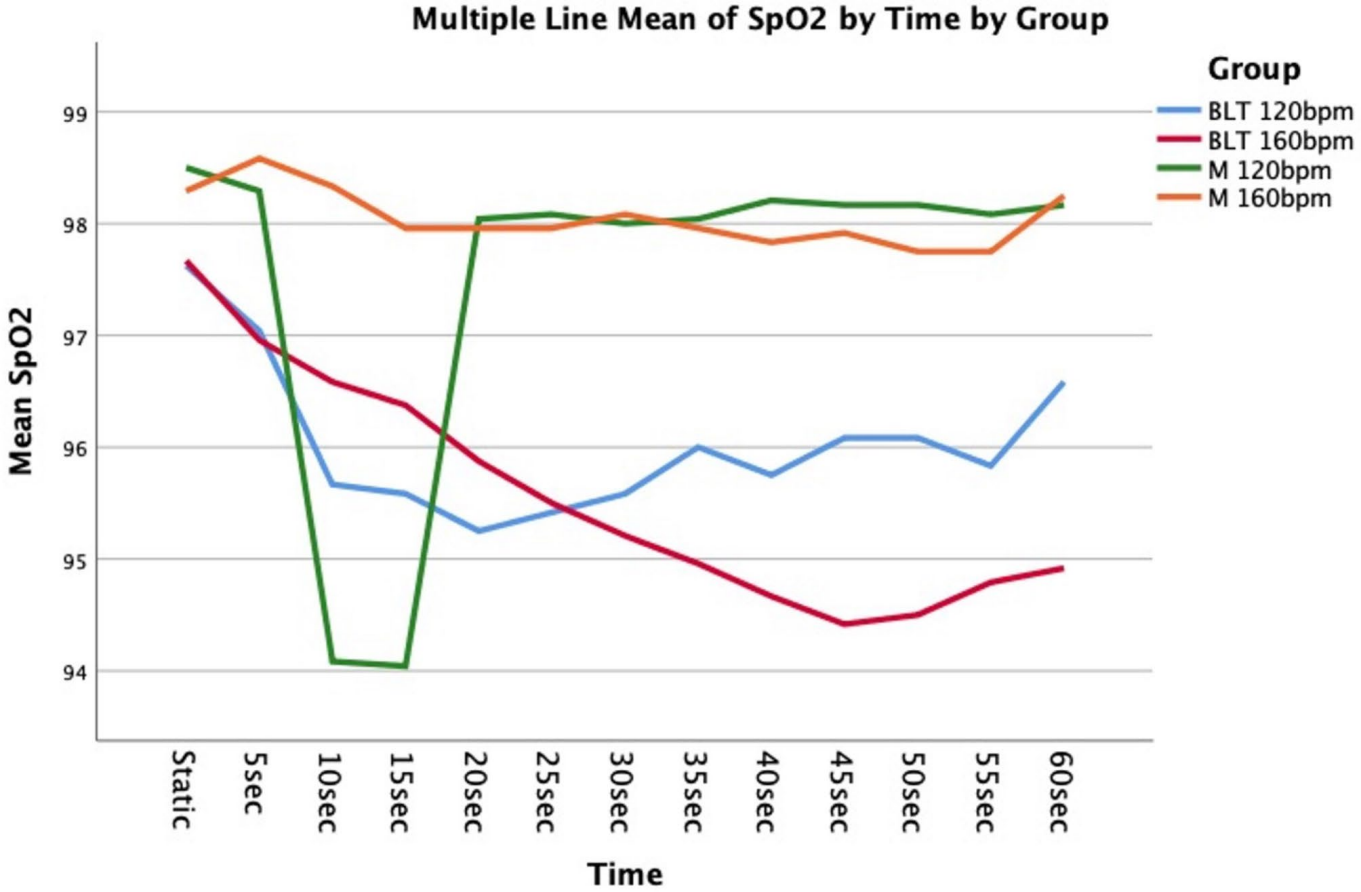
The graph shows the mean SpO_2_ readings over 60 s in 24 patients in 5-second intervals. Masimo SpO_2_ is abbreviated as Device A and Biolight Analog SpO_2_ is abbreviated as Device B. 120 bpm and 160 bpm were the two subgroups in the waving motion group and 2-tap motion groups

According to the results of the Angular Motion Experiment, in the 120 bpm subgroup, Device B’s SpO_2_ values remained stable between 95 and 98 throughout the experiment. In contrast, Device A’s SpO_2_ values dropped from 98.5 to 94% after 8 s but recovered to 98% after another 10 s. In the 160 bpm subgroup, Device B’s SpO_2_ values gradually decreased below 97–94.4%, whereas Device A’s SpO_2_ values remained stable at 98%. Device A showed slightly higher levels of waveform disruptions in both subgroups when compared to Device B. However, the difference was not significant. The waveform disruption was moderate in both. See Fig. 4.

### Waving motion group

Regarding the Technical and SpO_2_ Alarms, the results for the Waving Motion Group showed no alarms for both devices for the 60-second duration in both the 120 bpm and 160 bpm subgroups.

### 2-tap motion group

In the 2-Tap Motion Group, there were no alarms for the entire 60-second duration in the 120 bpm subgroup. However, in the 160 bpm subgroup of the 2-Tap Motion Group, in the first 20 s, Device A sounded 2 technical alarms compared to Device B, which sounded 1, whereas Device B sounded 4 SpO_2_ Alarms compared to Device A, which sounded 1. From 20 to 60 s, Device A sounded 6 technical alarms, whereas Device B sounded 17 SpO_2_ Alarms during this time. During this duration of 20–60 s, Device A did not sound any SpO_2_ alarms, whereas Device B did not sound any Technical Alarms. See Table 3.

**Table 3:** Angular motion experiment. Data is shown as mean ± standard deviation and sums (*n* = 24)

**Waving Motion Group**							
**Pace**	**Phase**	**Device A**^**1**^ **Waveform Disruption (0–4)**	**Device B**^**2**^ **Waveform Disruption (0–4)**	**Device A Technical Alarms (** ***n*** **)**	**Device B Technical Alarms (** ***n*** **)**	**Device A SpO**_**2**_ **Alarms (*n*)**	**Device B SpO**_**2**_ **Alarms (*n*)**
120 bpm	0–20 s	2.2 ± 0.4	2.2 ± 0.3	0	0	0	0
	20–40 s			0	0	0	0
	40–60 s			0	0	0	0
160 bpm	0–20 s	2.4 ± 0.4	2.0 ± 0.3	0	0	0	0
	20–40 s			0	0	0	0
	40–60 s			0	0	0	0
**2-Tap Motion Group**							
**Pace**	**Phase**	**Device A Waveform Disruption (0–4)**	**Device B Waveform Disruption (0–4)**	**Device A Technical Alarms (** ***n*** **)**	**Device B Technical Alarms (** ***n*** **)**	**Device A SpO**_**2**_ **Alarms (*n*)**	**Device B SpO**_**2**_ **Alarms (*n*)**
120 bpm	0–20 s	2.1 ± 0.6	2.0 ± 0.4	0	0	0	0
	20–40 s			0	0	0	0
	40–60 s			0	0	0	0
160 bpm	0–20 s	2.2 ± 0.5	2.1 ± 0.5	2	1	1	4
	20–40 s			4	0	0	10
	40–60 s			2	0	0	7

^1^Device A = Masimo Analog SpO_2_, ^2^Device B = Biolight Analog SpO_2_

## Discussion

The primary outcome of technical and SpO_2_ alarms showed no significant differences up in all experiment groups and subgroups except in the 160 bpm 2-tap motion experiment. The SpO2 values and waveform disruptions showed no significant differences. We conducted a total of three experiments with the objective of identifying the anti-motion resistance capabilities of Analog SpO_2_ Devices. We tested two devices for motion resistance and compared them to each other. The purpose of these experiments was to evaluate the clinical use of Analog SpO_2_ Devices under motion.

### The control experiment

According to the results of the first experiment, The Control Experiment, when there was no motion, there were no waveform disruptions, and no technical or SpO_2_ Alarms. This experiment proved that the setup was stable, and both devices performed nominally.

### Linear motion experiment

The Linear Motion Experiment was designed to test the effect of linear displacement over a fixed distance that a patient would move their hand in the hospital. The purpose of having a fixed defined distance and an accelerating slider on rails was to test the device up to its limits; however, with our testing, we were unable to reach the limits with this controlled movement. Initially, during our preliminary testing phase, we had planned to use a simulator to minimize the number of variables as we could fix the simulator to the camera slider. However, we realized that the setup wouldn’t work as the simulator did not produce any wave disruptions or changes in readings; it could not simulate a real human hand. Therefore, we decided to do the experiment with real human participants who volunteered to participate. With all the liner motion experimentation, we found that the Analog SpO_2_ readings of both devices were comparable, there were no alarms, and the waveforms of Device B were more stable than those of Device A; however, there was no significant difference. These experiments were quite challenging for both devices, yet both devices displayed anti-motion resistance capabilities.

### Angular motion experiments

From The Linear Motion experiments, we believe that these experiments, although they do represent a certain kind of hand motion, the actual motion of the hand might also involve some angular motion since the hand is attached to the forearm and pivots at the elbow, creating an angular motion. These experiments also proved that even with a rigorous continuous motion of up to 160 bpm, neither device sounded any alarms, and the readings were almost identical as long as the hand didn’t bump into anything, which is what we saw in The Waving Motion group. Even when it bumped into things, the readings were stable, and no alarms sounded at 120 bpm with both devices.

In these experiments, we collected the data in three phases: 0–20 s, 20–40 s, and 40–60 s. If a device started sounding the alarm in the first phase and ended in the last phase, that counted as 3 alarms instead of 1. In the 160 bpm experiment, Device A sounded more technical alarms, whereas Device B sounded more SpO_2_ alarms. In total, Device A sounded 8 technical alarms, while Device B sounded 1. Device A sounded 1 SpO_2_ alarm, whereas Device B sounded 21. It is to be noted that when Device A sounded technical alarms, it was not reading SpO_2_ values accurately. Therefore, the technical alarms, and thus it means that it could not sound SpO_2_ alarms during that period. Device B’s alarms could either be misreadings or an actual decrease in SpO_2_ due to decreased perfusion; it is hard to judge the exact reason for the alarms.

Most of Device B’s SpO_2_ alarms started sounding in the 20–40 s phase and ended either after the experiment ended or in the 40–60 s phase. This means that for the first 20 s, there were fewer alarms from both devices, which is what we believe exceeds the maximum duration of such continuous rigorous motion for a patient in the hospital. The 160 bpm 2-tap angular motion is very fast-paced, and we believe the reason for most of the alarms might not be the motion but the tapping on a hard surface. Tapping at such a high pace could misalign the sensor, causing it to sound alarms.

### Clinical implications

The findings of this study demonstrate that Analog SpO_2_ Pulse Oximetry exhibits motion resistance up to limits significantly higher than those encountered in a typical hospital or clinical setting during routine use. This suggests that this technology can be considered motion-resistant for daily clinical use. However, it is imperative for healthcare providers to maintain a nuanced understanding of these devices’ limitations, as occasional motion artifacts can still occur. Waveform disruptions generally do not trigger alarms, it is when the device is unable to monitor the SpO_2_ value of the patient, that it triggers an alarm. In our results Device A considered this a technical issue and Device B considered it an SpO_2_ alarm. There are essentially two types of alarms: technical or physiological. Technical alarms sound when there is a technical issue with the device, and it is unable to monitor the physiological parameter for any reason, which could be a faulty sensor, when the device detects motion, when the device detects too much disturbance in the signal, or other technical reasons. These alarms are generally low priority alarms and make less noise compared to physiological alarms. Physiological alarms such as SpO_2_ alarms sound when the device is able to monitor the physiological parameters nominally, however, it detects that the physiological measurement is above or below a certain limit that is defined by the user and generally has a default value set by the manufacturer. These alarms are designed with in accordance with standards set by the International Organization for Standardization (ISO), specifically IEC 60601-1-8:2006 standard [20]. When a technical alarm sounds, it means the patient is not being monitored for now due to a technical reason, this does not mean that an abnormal physiological event cannot occur at this time. Therefore, the patient needs to be monitored manually until the issue that caused the technical alarm is resolved. When a physiological alarm sounds, that means the patient’s physiological parameter has exceeded the defined limit and the healthcare person on duty must attend to the patient immediately. Regardless of the type of the alarm, when the alarm sounds, the physician or nurse on call should respond in a timely manner. However, many times some of these alarms are considered false alarms since they might be caused as a result of a technical issue. This analog technology however could potentially reduce the number of false alarms significantly.

### Minimizing alarm fatigue

Alarms triggered by motion artifacts can result in an overwhelming number of false alarms leading to many hospitals completely turning the alarms off or getting somewhat immune to the sound. Analog SpO_2_ technology could minimize the alarms caused by motion artifacts and allow healthcare providers to focus on alarms that matter. Improving Patient Safety and reliability of the device in the healthcare setting. Healthcare professionals should also read user manuals that come with the devices in order to make sure of the proper usage of the devices. Some things that can help reduce false alarms are making sure that the surface of the finger on which the sensor is attached to is not dirty, over-pigmented, tattooed, injured, nail polished, cold, elevated, wet, on the same side as the blood pressure cuff is on, too large or too small for the probe, excessively in motion, exposed to bright lights other than from the probe, tucked under the body, or have reduced blood perfusion. The exact instructions on the proper usage of the device can differ and therefore instructions for a specific device are often mentioned in the user manuals provided by manufacturers, which healthcare professionals should read carefully before use.

### Future research

The study highlights the need for further research into technologies and techniques that can further reduce the number of false alarms triggered by non-medical events. Future studies should also test these technologies on cardiac disease and/or respiratory disease patients.

### Limitations

Most alarms occurred when the subject’s hands were cold. We did not check the temperature of the hands before the experiment; after the experiment, we touched the hands of the participants and realized they were cold. However, we could not heat the hands and re-record the readings as this was not part of the design of the experiment. However, we do believe that perfusion could have been improved by heating the hands a little, and the results of the experiment might have had fewer alarms. Another limitation is the small sample size and single-center study; the results might have been more generalizable with more participants from different centers. However, the participants in this study did have different ethnic backgrounds with varying complexions of skin, as this has been shown to affect the results of pulse oximetry [21, 22]. Another weakness of this study is that it was only performed on adults, because of how hard it would have been for children to comply with since the instructions were quite complex. Lastly, this study only compared two technologies using one monitor. We contacted some other brands, but they didn’t seem very keen to lend us their equipment for testing.

## Conclusions

In conclusion, our study found that Analog SpO_2_ was motion resistant under static, controlled linear motion conditions, and continuous rapid angular motion of up to the pace of 120 bpm. However, alarms were noted with a pace of 160 bpm. These findings emphasize the importance of healthcare provider awareness regarding device limitations and the need for further research to enhance monitoring devices for improved clinical utility.

## Data Availability

The datasets generated and/or analyzed during the current study are not publicly available due to data format being unable to upload to repositories but are available from the corresponding author on reasonable request.

## Abbreviations

bpm: Beats per minute; musical beats for rhythm generated by a metronome phone application
Device A: Masimo Analog Pulse Oximeter
Device B: Biolight Analog Pulse Oximeter
SpO_2_: Pulse Oximetry obtained via Blood Oxygen Saturation
